# Demographic patterns of a widespread long-lived tree are associated with rainfall and disturbances along rainfall gradients in SE Australia

**DOI:** 10.1002/ece3.626

**Published:** 2013-06-05

**Authors:** Janet S Cohn, Ian D Lunt, Ross A Bradstock, Quan Hua, Simon McDonald

**Affiliations:** 1 Department of Forest & Ecosystem Science, University of MelbourneCreswick, Victoria, Australia; 2 School of Environmental Sciences, Charles Sturt UniversityAlbury, New South Wales, Australia; 3 Faculty of Science, University of WollongongWollongong, New South Wales, Australia; 4 Australian Nuclear Science and Technology Organisation, Lucas HeightsNew South Wales, Australia

**Keywords:** *Callitris*, climatic gradients, demography, disturbances, grazing, tree distribution

## Abstract

Predicting species distributions with changing climate has often relied on climatic variables, but increasingly there is recognition that disturbance regimes should also be included in distribution models. We examined how changes in rainfall and disturbances along climatic gradients determined demographic patterns in a widespread and long-lived tree species, *Callitris glaucophylla* in SE Australia. We examined recruitment since 1950 in relation to annual (200–600 mm) and seasonal (summer, uniform, winter) rainfall gradients, edaphic factors (topography), and disturbance regimes (vertebrate grazing [tenure and species], fire). A switch from recruitment success to failure occurred at 405 mm mean annual rainfall, coincident with a change in grazing regime. Recruitment was lowest on farms with rabbits below 405 mm rainfall (mean = 0–0.89 cohorts) and highest on less-disturbed tenures with no rabbits above 405 mm rainfall (mean = 3.25 cohorts). Moderate levels of recruitment occurred where farms had no rabbits or less disturbed tenures had rabbits above and below 405 mm rainfall (mean = 1.71–1.77 cohorts). These results show that low annual rainfall and high levels of introduced grazing has led to aging, contracting populations, while higher annual rainfall with low levels of grazing has led to younger, expanding populations. This study demonstrates how demographic patterns vary with rainfall and spatial variations in disturbances, which are linked in complex ways to climatic gradients. Predicting changes in tree distribution with climate change requires knowledge of how rainfall and key disturbances (tenure, vertebrate grazing) will shift along climatic gradients.

## Introduction

A current challenge in ecology is to determine the processes influencing species distributions, so shifts in distributions under climate change can be predicted. Many models identify empirical relationships between current distributions of species and climatic variables that are used to estimate future distributions of species under climate scenarios (Thomas et al. 2004; Araújo and New 2007). As they do not consider population dynamics, biotic factors, and disturbances, it is uncertain whether species will track predicted shifts in suitable climate space (Pearson and Dawson 2003; Guisan and Thuiller 2005; Keith et al. 2008). In the few studies where these factors have been measured, biotic factors and disturbance regimes were just as important as climate in determining distributions and potential range shifts (Miller and Halpern 1998; García et al. 1999; Gworek et al. 2007; Boulant et al. 2009). This highlights the need to include biotic factors and disturbances in studies identifying drivers of species distributions.

For widespread species, different processes are likely to control demographic patterns in different parts of their distributions (Gworek et al. 2007). Climate can directly affect species distributions through variations in rainfall and temperature patterns (Gworek et al. 2007). For example, establishment of woody species in low rainfall systems is highly dependent on infrequent periods of above average rainfall (Denham and Auld 2004; Castro et al. 2005; Holmgren et al. 2006a; Squeo et al. 2007), resulting in fewer opportunities for establishment than in high rainfall systems. The timing of rainfall may be more important in low than high rainfall systems, as soil moisture stress in summer is a primary cause of plant mortality, especially for small, shallow-rooted seedlings (Denham and Auld 2004; Holmgren et al. 2006b; Squeo et al. 2007; López et al. 2008).

Climate can also affect species distributions indirectly through disturbance regimes (Gworek et al. 2007), which may be influenced by land use, the distribution of which is also determined by climate (Dale 1997; Luck 2007). For example, high levels of livestock grazing in arid systems (Walker 1993; Bureau of Rural Sciences 2009) have contributed to aging populations of woody species in many regions (García et al. 1999; Meyer and Pendleton 2005; Auld and Keith 2009). By comparison, in higher rainfall systems, where a range of land uses are economically viable (Dale 1997), disturbance regimes may be more variable across space. For instance, livestock grazing levels are likely to be higher on farms than on other tenures, such as roadsides (Lunt 1995; Fensham 1998), which may act as refuges, especially for palatable woody species. While fire frequencies have declined in some agricultural systems, leading to an expansion of fire sensitive woody species (Belsky and Blumenthal 1997; Noble 1997; Noss et al. 2006), large patches of woodland may maintain a regime of regular fires (Lunt et al. 2012). Identification of the way in which such processes vary and interact along climatic gradients will provide insight into how consequent demographic effects are likely to be altered by climate change.

Demographic patterns of widespread and long-lived species, associated with climatic factors and disturbance regimes can be identified in large-scale surveys (Peters et al. 2007; Prior et al. 2011). The distribution of trees is especially valuable because their longevity tells us about past influences (Dale et al. 2001). Seedlings of trees often experience demographic “bottle necks” (Grubb 1977; Richardson and Bond 1991), because their recruitment is more sensitive to changes in climate and disturbances than the mortality of adults (Lloyd 1997; Hanson and Weltzin 2000). Changes in the distribution of climatic and disturbance regimes associated with the demographic patterns of a species will influence future species distributions (Thuiller et al. 2008).

**Figure 1:**
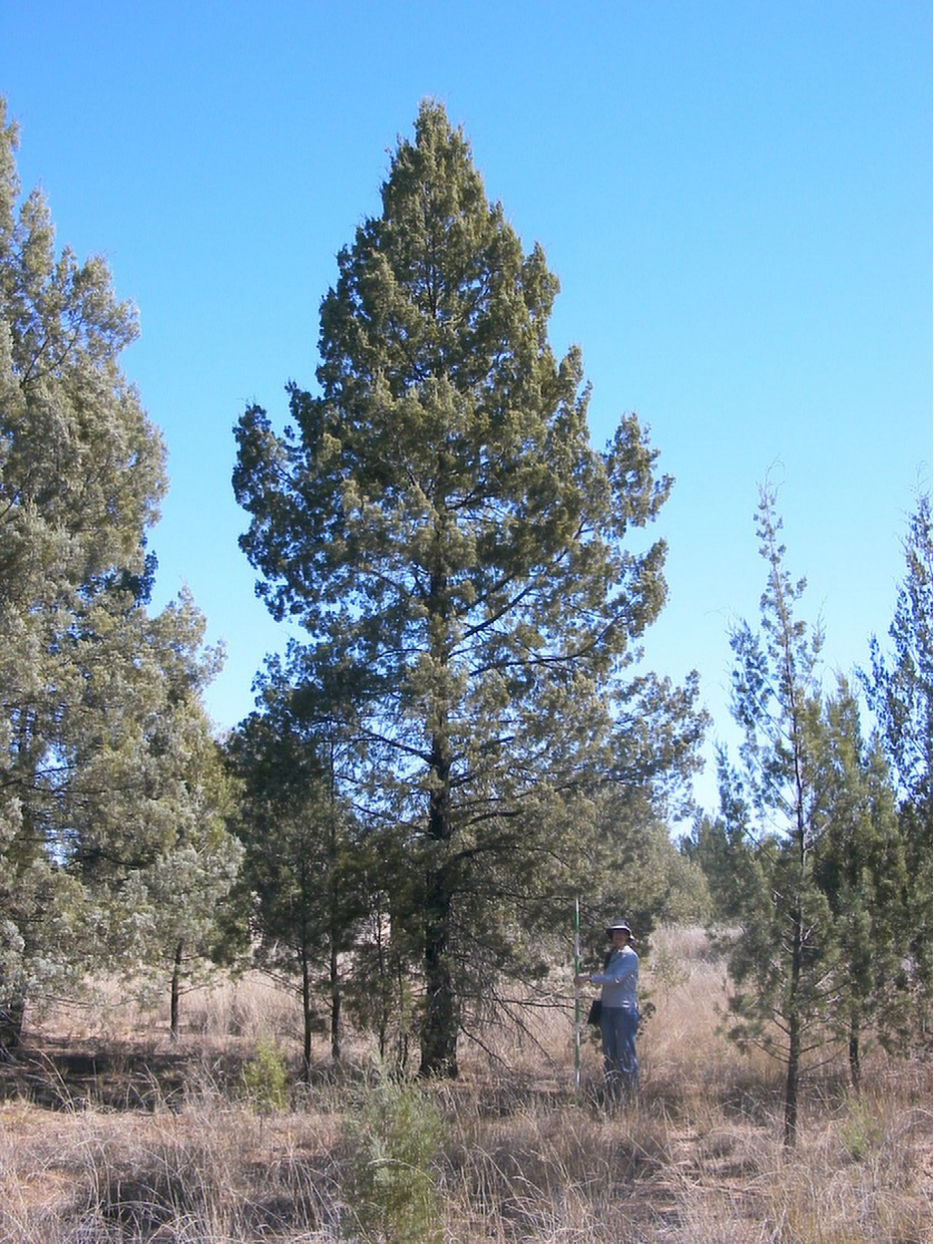
A *Callitris glaucophylla* tree (center), which established in the 1890s surrounded by more recent recruits in north eastern New South Wales, Australia.

Our aim was to examine how rainfall and disturbances along climatic gradients affect demographic patterns of a long-lived and widespread tree species, *Callitris glaucophylla* Thompson & Johnson (Fig. 1). This species, with a life span of at least 250 years and dominant in arid to temperate woodlands of Australia (Bowman et al. 1995; Prior et al. 2011), is ideal for examining how demographic processes in woody species may vary along climatic and disturbance gradients. *C. glaucophylla* is a slow-growing obligate-seeding conifer, which is sensitive to disturbances, as it does not resprout (Bowman et al. 1995). A number of studies have identified varying demographic patterns in *Callitris* species, which have been variously linked to climatic and disturbance variables. In a continental-scale survey of *C. columellaris,* recruitment was successful in tropical and temperate climates but absent in arid climates and linked to domestic stock grazing (Prior et al. 2011). In smaller scale studies, recruitment of *Callitris* species was associated with above average rainfall and absent under the influence of grazing by sheep or cattle and high intensity fires (Austin and Williams 1988; Bowman and Latz 1993; Noble 1997; Prior et al. 2010; Russell-Smith et al. 2012). No studies have aged *C. glaucophylla* individuals to determine relationships between recruitment, rainfall events, and disturbance regimes across climatic gradients. Relationships between these factors are likely to govern the way that the distribution of *C. glaucophylla* and other woody species will change in the future under climate change.

In this study, we explored the way that rainfall and disturbances interact at macro-scales across the distribution of *C. glaucophylla*. We hypothesized: (1) disturbance regimes will vary along rainfall gradients; (2) in the absence of inter- and intra-specific competition, fewer cohorts are more likely where annual rainfall is low and winter dominant, especially where disturbances are frequent; and (3) more cohorts are expected where annual rainfall is higher, especially where disturbances are less frequent. We then use these insights to predict future demographic and distributional changes in *C. glaucophylla* populations across south eastern Australia in response to climate change.

## Methods

**Figure 2:**
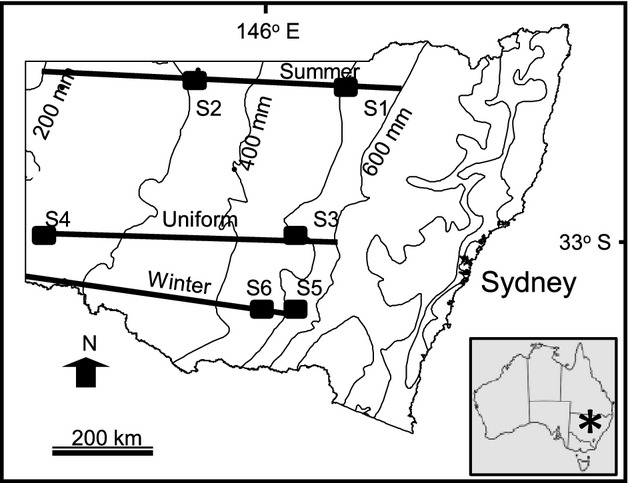
The location of transects used to survey *Callitris glaucophylla* recruitment in New South Wales, Australia. Transects follow gradients of declining mean annual rainfall (200–600 mm) from east to west and are located in seasonal rainfall zones from summer in the north, uniform in the middle to winter in the south. Sites where wood samples were taken for radiocarbon dating are marked from S1 to S6. Isohyets are labeled with the mean annual rainfall.

Stands of *C. glaucophylla* were sampled along three transects of declining mean annual rainfall and varying seasonal rainfall dominance in New South Wales, Australia (Colls and Whitaker 2001; Bureau of Meteorology 2010; Fig. 2). Transects extended from the arid zone in the west (200 mm annual rainfall) to the temperate zone in the east (600 mm annual rainfall; Specht and Specht 1999; Bureau of Meteorology 2006). Rainfall varies seasonally from north to south with summer dominant rainfall in the north (60% excess in summer cf. winter, Lacey 1973), uniform rainfall in the middle and winter dominant rainfall in the south (40% excess rainfall in winter cf. summer). Hereafter, transects will be referred to as the summer (29.36°S 149.76°E to 29.03°S 141.44°E), uniform (33.15°S 148.17°E to 32.77°S 141.63°E), and winter rainfall zones (34.80°S 147.20°E to 34.01°S 141.05°E). Each transect was approximately 600 km in length. Fieldwork was undertaken from May to August 2008.

Sites were chosen to sample the maximum number of cohorts at intervals along each transect. In the higher rainfall area to the east, sites were on average 30 km apart, however, in the lower rainfall areas in the west, potential sites were less frequent, and previous vegetation surveys were used to identify sites within 50 km north or south of each transect (Fox 1991; Scott 1992; Porteners 1993; Porteners et al. 1997; Keith 2004; Benson et al. 2006). A total of 56 sites were sampled including 16 in the summer, 23 in the uniform and 17 in the winter rainfall zone.

A prerequisite for site inclusion was the presence of mature *C. glaucophylla* trees (late-19th century recruitment; Lacey 1972) as a seed source. This cohort established before rabbits reached large numbers from approximately 1879–1897 and there was little *C. glaucophylla* regeneration until rabbit numbers were significantly reduced in the mid 20th century (Thompson and Eldridge 2005). Other prerequisites included gaps between the canopies of the mature *C. glaucophylla* trees with at least 10% bare ground to provide space for potential recruitment, flat to undulating topography, no evidence of earthworks, logging, or fires since 1950s and minimal stock grazing. Where possible, fenced roadsides and state forests were sampled, since these were generally less intensely grazed than travelling stock routes and paddocks (Fensham 1998; Lunt 2005). However, there were few less disturbed sites in low rainfall areas in the west, where roadsides were often unfenced from adjacent farms, and state forests were less common.

At each site a number of variables were measured within a 0.1 ha quadrat. The number of cohorts, which had established since 1950s was counted. Cohorts were distinguished by differences in height and canopy shape, presence of lower stems, lichens on bark, and local knowledge of land managers (Read 1995; Whipp 2009). Cohorts were allocated to a relative age category from A to D, with A being the oldest and D the youngest at each site. Where there was uncertainty in differentiating between two cohorts they were grouped into the one category, resulting in a higher probability of underestimating than overestimating cohort numbers. The height and diameter of the two tallest individuals from each cohort were measured.

Bomb-pulse radiocarbon (^14^C) dating was used to age *C. glaucophylla* individuals (Hua 2009; Pearson et al. 2011). Radiocarbon dating of representative basal disks of *C. glaucophylla* individuals from two sites along each transect (distant where possible) was used to confirm recruitment dates assessed by ring counting (Hua 2009; Fig. 2). This involved dating one wood specimen from each cohort at four sites (winter and uniform) and duplicate wood specimens from each cohort at two sites (summer) to examine within site reliability of cohort assessment. Disks from the younger trees were removed near ground level and disks from the oldest trees were removed 20 cm from the ground. Radiocarbon dating on the third ring from the pith of each sample was carried out using the STAR accelerator mass spectrometry facility at Australian Nuclear Science and Technology Organisation (Hua et al. 2001; Fink et al. 2004). As the precision of radiocarbon dating is low for the most recent recruits (≥2003), they were dated by ring counting only.

Recent grazing was assessed by recording the presence of native macropods (Macropodidae) and introduced vertebrates including cattle (*Bos taurus* L), sheep (*Ovis aries* L), European rabbits (*Oryctolagus cuniculus* L), and goats (*Capra hircus* L). Herbivores were either sighted directly or indirectly by scats, warrens or diggings. Evidence of fire since 1950s was based on charred stems or stags of trees that established after 1950. Land tenure was used as a surrogate for longer term grazing intensity, measured as low to moderate and high: low to moderate for non-farms, including roadsides fenced from adjacent farms, travelling stock routes, state forests, and national parks; high for farms and adjacent unfenced roadsides.

### Statistical analyses

We used regression tree analyses to examine the suite of variables associated with the number of cohorts at each site (Table 1). Overfitting was avoided by pruning the tree to minimize the cross-validated error (Venables and Ripley 2002). Categorical variables which occurred in fewer than 30% of sites were excluded from the analyses (e.g., fire). Spearman's rank coefficient was used to examine collinearity between remaining pairs. In the first regression tree analysis all explanatory variables with |*r*| ≤ 0.5 were included (Tabachnick and Fidell 2007), i.e., annual rainfall (mm), latitude, topographic position (flat, dune), land use (non-farm, farm), and the presence of rabbits, sheep, cattle, goats, and kangaroos. In the second regression tree analysis, annual rainfall was removed, as a number of explanatory variables were significantly correlated with annual rainfall including land use (*r* = −0.46, *P* < 0.001), rabbits (*r* = −0.48, *P* < 0.001), goats (*r* = −0.34, *P* < 0.01), and topographic position (*r* = −0.34, *P* < 0.01). No other explanatory variables were significantly correlated. All analyses were undertaken in SPLUS software version 8 for Windows (Insightful Corp 2007).

**Table 1:** A description of the variables used in the regression tree analyses and how they were assessed

Variable	Values	How assessed
Annual rainfall (mm yr^−1^)	200–600	Bureau of Meteorology (2006)
Seasonal rainfall	Summer, uniform, winter	Bureau of Meteorology (2010)
Land use type	Fenced roadside (l-m)	Maps/Observations
	State forest (l-m)	
	National park (l-m)	
	Travelling stock route (l-m)	
	Unfenced roadside (h)	
	Farm (h)	
Herbivores (rabbits, sheep cattle, goats, kangaroos)	Present/Absent	Sighting scats, warrens, diggings
Fire after 1950	Present/Absent	Charred post-1950 *Callitris*

Land use type indicated long-term grazing intensity, which was rated low-moderate (l-m) or high (h). BOM is the Bureau of Meteorology.

On the basis of the results from the regression tree analysis, we examined trends in the number of cohorts with annual rainfall in each seasonal rainfall zone, by fitting linear, loess, and sigmoidal relationships. Curves of best fit were determined using the residual sum of squares (RSS) and Akaike Information Criterion (AIC), with lower values of either indicating a better fit (Quinn and Keough 2002). A goodness of fit was calculated for each model using *R*^2^ = RSS/Total Sum Squares (TSS).

**Figure 3:**
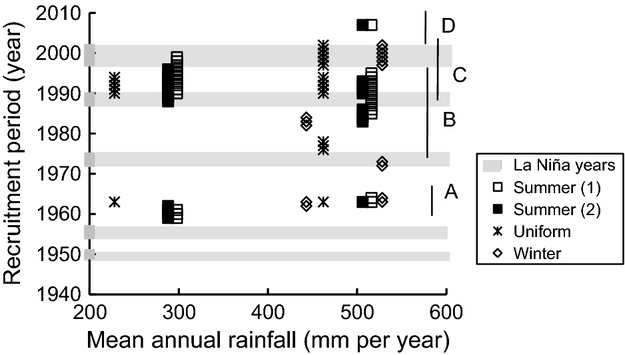
Recruitment periods of *Callitris glaucophylla* specimens, derived from radiocarbon dating versus mean annual rainfall in each seasonal rainfall zone (summer 1 = duplicate 1, summer 2 = duplicate 2, uniform, winter). Cohorts identified in the field are indicated by A, B, C, or D and represented by vertical lines. Cohort A is likely to be older than the dating indicates, as disks were taken at least 20 cm above the ground. Periods of La Niña are also shown.

We examined the potential opportunities for *C. glaucophylla* regeneration since 1950 by deriving a rainfall index. This index was derived at each site by assuming that *C. glaucophylla* regeneration is dependent on high rainfall in summer, a well documented phenomenon (Forestry Commission of New South Wales 1988). First, we defined the summer rainfall (December–February) value associated with the estimated time of establishment for each radiocarbon dated sample (Fig. 3). We then selected the lowest summer rainfall estimate from the values defined in the first step and assumed that this equated to the minimum amount of summer rain needed for establishment. Finally, we estimated the number of summers from 1950 to 2008 that this minimum value was reached at each site, and from this we constructed an index of potential opportunities for *C. glaucophylla* regeneration along each seasonal rainfall transect. The higher the index, the greater the potential opportunities for *C. glaucophylla* regeneration. We examined the effects of mean annual rainfall, seasonal rainfall (summer, uniform, winter) and their interaction on the index of potential opportunities for *C. glaucophylla* regeneration from 1950, using an Analysis of Covariance (ANCOVA). Data were square root transformed to satisfy homogeneity of variances. Post hoc pairwise comparisons used Bonferroni correction.

We used non-parametric proportion tests to examine the proportional occurrence of disturbances, namely farms, rabbits, sheep, cattle, goats, and kangaroos with mean annual rainfall (<405 mm, ≥405 mm) and seasonal rainfall zone (summer, uniform, winter). Evidence of fires was too infrequent to analyze. Post hoc tests used the Bonferroni correction.

## Results

While field recognition of discrete cohorts within sites was reliable, recognition of the same age class between sites was not always reliable (Appendix 1). The oldest cohort (Cohort A) was consistently dated from 1959 to 1964 by radiocarbon, although it is likely that these established in the 1950s (Thompson and Eldridge 2005), as samples were cut 20 cm above the ground, giving an underestimate of their ages (Fig. 3). Cohort B was considered older than C in the field, however, dating showed overlap between sites. Cohort B dated from 1972 to 1996 and cohort C from 1988 to 2002. This agreed with the ring counts, which also indicated that Cohort D, which established from 2003 to 2006 was the youngest. In five of the six cases where replicate wood specimens were dated within a site, radiocarbon results of these specimens delivered the same recruitment year. On the one occasion where this did not occur, there was only 1 year difference between the duplicate specimens.

**Figure 4:**
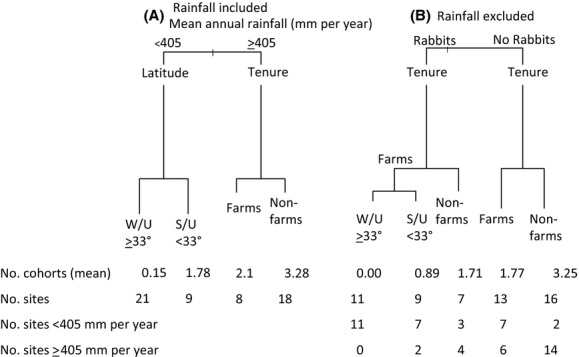
Results from regression tree analyses showing the factors associated with the average number of cohorts since 1950, when mean annual rainfall was (A) included and (B) excluded. In (B) the number of sites less than (<) and greater than or equal to (≥) 405 mm mean annual rainfall is given below each branch. Seasonally dominant rainfall is in summer (S), uniform (U), and winter (W).

The first regression tree analysis indicated that cohort number was associated with annual rainfall, seasonal rainfall (i.e., latitude), and land tenure (adjusted *R*^2^ = 0.65, Fig. 4A). The root or primary division in the regression tree occurred at 405 mm mean annual rainfall. There were fewer cohorts below 405 mm mean annual rainfall, especially in the winter rainfall zone compared with the summer rainfall zone (0.15 *c.f*. 1.78). Above 405 mm mean annual rainfall, farms had fewer cohorts than less disturbed tenures, such as roadsides, travelling stock routes, state forests, and national parks (2.13 *c.f*. 3.28).

The second regression tree analysis, which excluded mean annual rainfall, indicated that cohort number was associated with rabbit occurrence and land tenure (adjusted *R*^2^ = 0.65, Fig. 4B). The mean number of cohorts was lowest on farms with rabbits in the winter and uniform rainfall zones (0) and marginally higher in the summer and uniform rainfall zones (0.89). Most of these sites occurred below 405 mm mean annual rainfall. The highest number of cohorts occurred where there were no rabbits on less disturbed tenures, namely roadsides, travelling stock routes, state forests, and national parks (3.25). Most of these sites occurred above 405 mm mean annual rainfall. Moderate numbers of cohorts were on farms without rabbits (1.77) and less disturbed tenures with rabbits (1.71). These sites were distributed equally above and below 405 mm mean annual rainfall.

**Figure 5:**
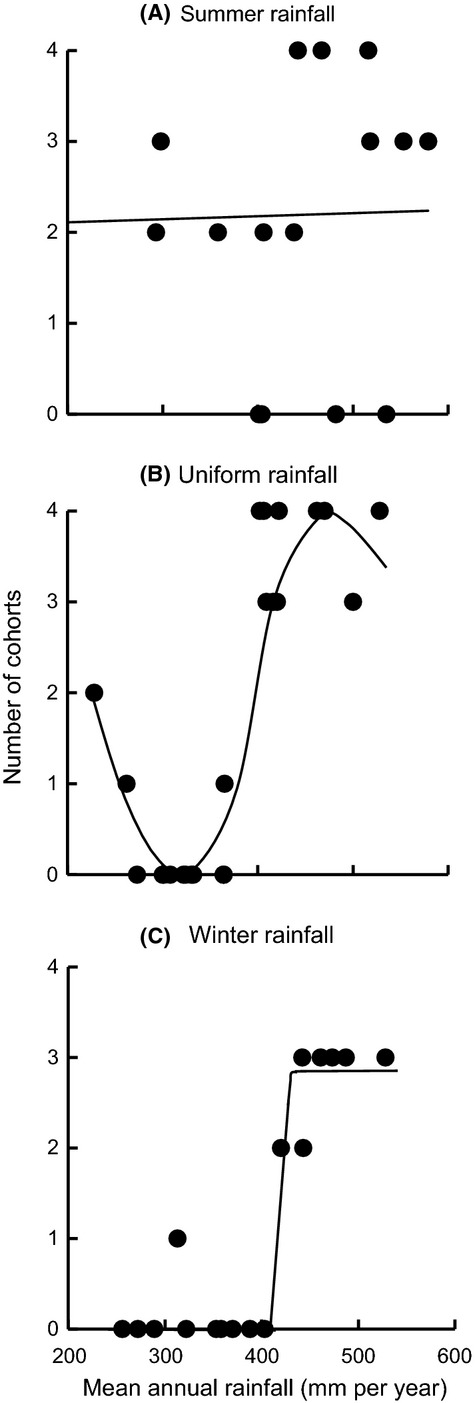
Curves of best fit (based on AIC) for the number of cohorts since 1950 versus mean annual rainfall in each seasonal rainfall zone.

Curve fitting identified varying trends in the number of cohorts with mean annual rainfall, depending on the seasonal rainfall zone (Fig. 5). Abrupt declines in the number of cohorts at approximately 405 mm annual rainfall in the winter and uniform rainfall zones were best described by a sigmoid and loess curve, respectively, based on low values for RSS and AIC (*R*^2^ = 0.94, 0.92; Appendix 2). In the summer rainfall zone, there was little relationship between cohort number and annual rainfall using linear or loess curves (*R*^2^ = 0.18, 0.001; Appendix 2).

**Figure 6:**
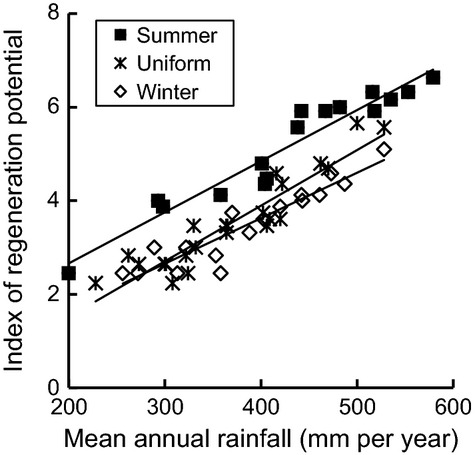
Linear trends in an index of potential opportunities for *Callitris glaucophylla* regeneration in summer from 1950 to 2008 versus mean annual rainfall in each seasonal rainfall zone (summer, uniform, winter).

The index of potential opportunities for *C. glaucophylla* regeneration (square root transformed) since 1950, was significantly and positively associated with mean annual rainfall treated as a continuous variable (*P* < 0.001) and significantly associated with seasonal rainfall (summer, uniform, winter (*P* < 0.001)), but not their interaction (*P* = 0.31) using ANCOVA (Fig. 6, Appendix 3). Post hoc pairwise tests indicated that the index was significantly greater in summer than either the uniform or winter rainfall zones, but not significantly different between the winter and uniform rainfall zones.

**Figure 7:**
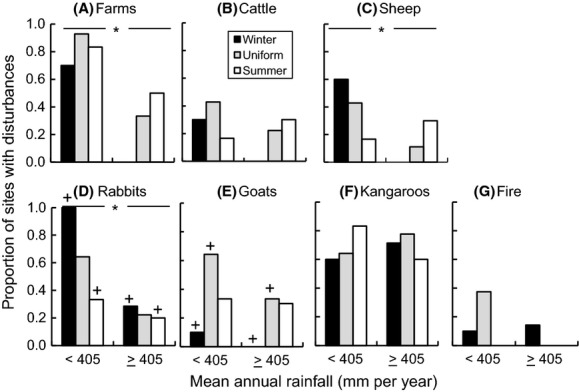
Proportion of sample sites with farms, grazing species, and fire versus mean annual rainfall (< and ≥405 mm) in each seasonal rainfall zone (summer, winter, uniform). Asterisks and pluses indicate significant differences between < 405 and ≥405 mm mean annual rainfall and seasons, respectively. As few sites were burned, fire was not statistically analyzed.

Proportion tests were used to examine the occurrence of disturbances with mean annual rainfall and seasonal rainfall (Fig. 7, Appendix 4). A significantly greater proportion of sample sites contained farms (*P* = 0.002), rabbits (*P* = 0.001), and sheep (*P* = 0.048), below 405 mm mean annual rainfall than above. The proportion of sites with farms (*P* = 0.190), sheep (*P* = 0.810), cattle (*P* = 0.480), and kangaroos (*P* = 0.480) was not significantly different between the seasonal rainfall zones. The proportion of sites with rabbits was significantly greater in the winter than the summer rainfall zone (*P* = 0.030), and for goats was significantly greater in the uniform than the winter rainfall zone (*P* = 0.008). Although not statistically analyzed, a greater proportion of fires occurred at less than 405 mm mean annual rainfall than above.

## Discussion

These results indicate major differences in *C. glaucophylla* recruitment patterns across the species’ range in western New South Wales. Since 1950s, *C. glaucophylla* has recruited frequently in high-rainfall areas (≥405 mm mean annual rainfall), and in areas with summer-dominated rainfall. In contrast, most populations in low rainfall areas (<405 mm) with winter-dominated rainfall have experienced no recruitment since the late 1800s (Thompson and Eldridge 2005). At the scale of the study, disturbance regimes were not independent of climatic patterns. Consequently, recruitment patterns were closely associated with regional patterns of disturbance and land use, especially grazing by livestock, feral, and native mammals. *C. glaucophylla* recruited more frequently on tenures that were less frequently grazed by livestock and in areas with no evidence of recent grazing by feral rabbits.

### Rainfall and recruitment

Many studies have reported infrequent, pulsed recruitment of woody plants in arid and semi-arid regions (Watson et al. 1997b; Holmgren and Scheffer 2001; Wiegand et al. 2004; Holmgren et al. 2006b; Prior et al. 2011). Inadequate soil moisture in most years prevents seedling establishment and causes high mortality of young plants (Watson et al. 1997a; Holmgren et al. 2006a; Gutiérrez et al. 2007). *C. glaucophylla,* however, is a long-lived tree with a life-span of over 200 years (Bowman et al. 1995). Consequently, an absence of recruitment for a century does not necessarily indicate a decline in long-term population viability, as rare recruitment events may enable populations to persist (e.g., Wiegand et al. 2004). However, tree mortality was apparent at some sites, indicating that populations may have declined in recent decades.

Additionally, historical rainfall records indicate that the winter-rainfall region has experienced a number of high rainfall (La Niña) events over the past century, especially in 1948–1949, 1955–1956, 1973–1974, 1988–1989, and 1998–2000 (Nicholls et al. 1992; Bureau of Meteorology 2005). Rainfall in these years was well above the mean annual rainfall or in the top decile rank (Bureau of Meteorology 2005). These events triggered widespread recruitment of woody plants including *C. glaucophylla* in SE Australia (Austin and Williams 1988; Harrington 1991; Denny 1992; Read 1995; Noble 1997; Parker and Lunt 2000), but only limited recruitment of woody species in arid and semi-arid regions (Hall et al. 1964; Chesterfield and Parsons 1985), and sometimes only within fenced areas (Hall et al. 1964). Consequently, the absence of *C. glaucophylla* recruitment during the 1900s from this low rainfall region (especially winter-rainfall region) is unlikely to be due to inadequate rainfall. Indeed, other authors have attributed declines of woody species in this region to unfavorable disturbance regimes, particularly grazing (Auld and Keith 2009; Prior et al. 2011).

In contrast, *C. glaucophylla* has recruited frequently over the past 60 years in high rainfall areas (>405 mm mean annual rainfall), consistent with trends in a continental-scale survey by Prior et al. (2011). For the first half of the 20th century, *C. glaucophylla* populations contained plants derived from the late 1800s and earlier, with no younger cohorts (Lacey 1972; Austin and Williams 1988). Repeated recruitment since1950s means that population density has increased (Lacey 1972; Austin and Williams 1988) and mean population age has declined. *C. glaucophylla* stands self-thin extremely slowly (Lacey 1972; Forestry Commission of New South Wales 1988). Consequently, these fire-sensitive stands are becoming increasingly dense as recruitment continues. A current lack of high intensity fires, prevalent before agriculture and land fragmentation (Noble 1997; Noble and Dargavel 2001) also promotes these changes (Ross et al. 2012). This phenomenon is similar to that found in fire-sensitive *Pinus ponderosa* populations in the long-term absence of fire in mixed *Pinus*-*Quercus* forests in the western U. S. A. (Noss et al. 2006).

Seasonal rainfall had a strong effect on the number of *C. glaucophylla* cohorts, with establishment being particularly restricted in low rainfall areas (<405 mm) in winter as opposed to summer-dominated rainfall areas. In dry regions, soil moisture stress in summer is a primary cause of plant mortality, especially for small, shallow-rooted seedlings (Denham and Auld 2004; Holmgren et al. 2006b; Squeo et al. 2007; López et al. 2008). Consequently, for a given amount of annual rainfall, summer or uniform rainfall supports greater plant productivity than winter rainfall (Holmgren et al. 2006b; Lopez et al. 2006; Squeo et al. 2007). The coincidence of seed shedding in summer by *C. glaucophylla* (Lacey 1972) and summer rainfall may also increase opportunities for establishment in the summer-dominated rainfall zone.

### Land use, herbivory, and recruitment

Land use typically changes with climate, with greater agricultural productivity in higher rainfall areas, and extensive (“rangeland”) livestock grazing in low rainfall zones (Walker 1993). In this study, fencing patterns which control livestock movements varied across the rainfall gradient. In low rainfall areas, extensive rangeland grazing occurs, and relatively few roadsides or patches of remnant vegetation are fenced to prevent access by livestock. In contrast, in high rainfall areas, fences separate all paddocks from roadsides, and most patches of remnant vegetation on public land area are fenced. Consequently, *C. glaucophylla* stands on roadsides in high rainfall areas are less likely to be frequently grazed by livestock than stands on roadsides in semi-arid areas. This may explain why the number of *C. glaucophylla* cohorts was consistently greatest in “non-farm” land tenures (including roadsides and public land reserves) in areas of high rainfall.

Rabbits and sheep cause recruitment failure in *C. glaucophylla* and other woody species (e.g., *Acacia, Casuarina* spp.) as indicated by exclosure experiments and studies of population structure in semi-arid and arid Australia (Crisp and Lange 1976; Crisp 1978; Lange and Graham 1983; Chesterfield and Parsons 1985; Auld 1995; Tiver and Andrew 1997; Auld and Denham 2001; Prior et al. 2011). Similarly, our results suggest that the paucity of *C. glaucophylla* recruitment in semi-arid areas with winter and uniform rainfall is likely to be strongly influenced by grazing patterns. These trends are consistent with studies on woody shrubs in semi-arid regions elsewhere in the world (Bowers 1997; Gutiérrez et al. 2007; Holmgren et al. 2006b).

Our results highlight complex interactions among recruitment, disturbance regimes, and climate (Pearson and Dawson 2003; Peters et al. 2008). At a regional scale, variation in land use can provide refuges from livestock grazing and influence the abundance of feral animals. At a patch scale, grazing by livestock and feral animals directly influences establishment of woody plants. Broad-scale climate variation (e.g., high vs. low, and winter vs. summer rainfall) also affects these processes as shown by the variations in tree recruitment over a 60-year time-frame in this study. Future trends in recruitment will therefore depend on the way changes to management and climate interact (Fig. 7; Pearson and Dawson 2003; Guisan and Thuiller 2005; Keith et al. 2008).

### Future trends

Under climate change, temperatures are expected to increase in the study area in the future (Commonwealth Scientific and Industrial Research Organisation & Bureau of Meteorology 2007; IPCC et al. 2007; Hennessy et al. 2008). Although future rainfall patterns are less certain, projections indicate an increase in summer rainfall in the north of the region, strengthening existing patterns of summer dominant rainfall (Commonwealth Scientific and Industrial Research Organisation & Bureau of Meteorology 2007; Hennessy et al. 2008). In contrast, in southern areas, winter rainfall is expected to decline substantially, with only a slight increase in summer rainfall (Commonwealth Scientific and Industrial Research Organisation & Bureau of Meteorology 2007; Hennessy et al. 2008).

While infrequent high rainfall events may continue to drive major recruitment pulses, as has occurred in the past (Austin and Williams 1988), increasing aridity is likely to further restrict opportunities for *C. glaucophylla* recruitment, especially in dry, winter-rainfall areas. Thus, projected climatic changes are likely to intensify rather than ameliorate recruitment problems currently experienced in dry winter-rainfall areas. In contrast, the “leading edge” of the species distribution in high rainfall areas may expand eastward into adjacent *Eucalyptus*-dominated woodlands, in which *C. glaucophylla* is currently absent or rare, particularly if low fire frequency is maintained by land management practices.

A recent projection of climate change impacts on *Callitris* dominated woodlands in Western Australia based on bioclimatic envelope modeling suggested a major range expansion with no decline in areas occupied (Prober et al. 2012). In contrast, our results suggest that climate change is likely to intensify stresses on patches in dry winter-rainfall areas, potentially leading to range contractions in this sub-region. However, we emphasize that our results reflect historical inter-dependencies among land management practices, and these may not be continued in the future. In particular, recruitment opportunities and population viability may increase if greater control is exerted over grazing by feral animals and livestock in dry, winter-rainfall populations. If current grazing regimes persist or intensify, there appears to be a bleak future for *C. glaucophylla* populations and other woody species in this region regardless of climate change. This uncertainty may be resolved by experimentally manipulating grazing regimes in this sub-region.

## Conclusion

Demographic patterns in the widespread and long-lived tree species, *C. glaucophylla*, were closely associated with rainfall gradients and disturbance regimes. Disturbance regimes were associated with rainfall through different land use types. As climate changes, rainfall, land use, and disturbance regimes will shift along climatic gradients, influencing species distributions. To predict changes in the distribution of *C. glaucophylla* and other species, a greater understanding is required of how land management, disturbance regimes, and climate interact. Prediction of the outcome of novel combinations of these factors in the future will be challenging.

## Appendix 1

Radiocarbon dating results for *Callitris glaucophylla* basal disks.

**Table:**

					Percent modern carbon (pMC)		Calibrated ages at 95% confidence level	
						
	Lab ID	Sample ID	Transect	δ^13^C (‰)	Mean	1σ	Age range (AD)	Probability
1	OZL812	s2 1950/1	Summer	−22.9	117.09	0.45	***1959.64–1961.76***	0.14
							1987.33–1992.56	0.86
2	OZL813	s2 1950/2	Summer	−23.0	119.10	0.44	***1959.68–1962.4***	0.47
							1985.32–1990.12	0.53
3	OZL814	s2 1970/1	Summer	−23.8	114.16	0.40	1959.07–1959.64	0.16
							***1990.18–1994.34***	0.83
4	OZL815	s2 1970/2	Summer	−24.4	113.12	0.39	1958.77–1959.62	0.13
							***1992.36–1996.29***	0.85
5	OZL816	s2 1990/2000/1	Summer	−24.9	111.34	0.42	1958.72–1959.09	0.18
							***1995.15–1999.99***	0.81
6	OZL817	s2 1990/2000/2	Summer	−25.2	114.66	0.43	1959.09–1959.65	0.12
							***1988.46–1993.94***	0.88
7	OZL818	s1 1950/1	Summer	−22.6	146.13	0.65	***1963.78–1964.17***	0.07
							1972.27–1973.62	0.93
8	OZL819	s1 1950/2	Summer	−23.2	138.41	0.56	***1963.51–1963.91***	0.09
							1974.37–1976.24	0.91
9	OZL820	s1 1970/1	Summer	−22.6	119.71	0.47	1959.92–1962.43	0.52
							***1985.26–1989.33***	0.47
10	OZL821	s1 1970/2	Summer	−23.9	121.96	0.49	1961.84–1962.5	0.12
							***1983.11–1986.43***	0.88
11	OZL822	s1 1990/2000/1	Summer	−24.0	113.36	0.44	1959.04–1959.62	0.15
							***1990.36–1995.48***	0.84
12	OZL823	s1 1990/2000/2	Summer	−23.3	114.47	0.42	1959.09–1959.64	0.13
							***1990.17–1993.94***	0.86
13	OZL824	s4 1950/2	Uniform	−24.1	134.31	0.48	***1963.42–1963.61***	0.07
							1976.56–1978.17	0.93
14	OZL825	s4 1970/2	Uniform	−23.8	114.11	0.45	1959.07–1959.64	0.17
							***1990.18–1994.36***	0.82
15	OZL826	s3 1950/4	Uniform	−23.2	137.76	0.43	***1963.5–1963.89***	0.08
							1974.42–1976.25	0.92
16	OZL827	s3 1970/1	Uniform	−22.8	134.09	0.50	1963.4–1963.59	0.06
							***1976.57–1978.79***	0.94
17	OZL828	s3 1990/2000/2	Uniform	−22.8	114.26	0.44	1959.07–1959.64	0.15
							***1990.18–1994.33***	0.84
18	OZL829	s6 1950/2	Winter	−23.1	126.18	0.51	***1962.56–1963.26***	0.16
							1980.4–1982.79	0.83
19	OZL830	s6 1970/2	Winter	−23.9	123.59	0.42	1962.25–1962.72	0.05
							***1982.09–1984.67***	0.95
20	OZL831	s5 1950/1	Winter	−23.8	145.69	0.53	***1963.76–1964.17***	0.07
							1972.42–1973.64	0.93
21	OZL832	s5 1970/1	Winter	−22.8	145.33	0.54	1963.76–1964.2	0.08
							***1972.43–1973.65***	0.92
22	OZL833	s5 2000/2	Winter	−23.1	109.24	0.41	1958.64–1958.72	0.03
							***1997.45–2002.87***	0.97

Age calibration was performed using bomb ^14^C data for the Southern Hemisphere (Hua and Barbetti 2004) and the CALIBomb program (Reimer et al. 2004)

The likely calibrated ages are shown in bold italic.

## Appendix 2

Results of fitting curves to the number of cohorts with mean annual rainfall in each seasonal rainfall zone according to residual sum of squares (RSS), Akaikie Information Criterion (AIC), and *R*^2^.

**Table:**

Rainfall zone	Curve	RSS	AIC	*R*^2^
Summer	Linear	32.96	15.56	0.18
	Loess	26.96	20.40	0.001
Uniform	Loess	5.42	−21.43	0.92
	Sigmoid	7.40	−18.08	0.89
	Linear	29.53	9.75	0.57
Winter	Sigmoid	1.83	−29.86	0.94
	Loess	2.20	−22.87	0.93
	Linear	9.24	−6.36	0.70

Within each rainfall zone, models are ordered from best to worst using AIC.

## Appendix 3

Results of ANCOVA used to examine the effects of mean annual rainfall (continuous) and seasonal rainfall (summer s, uniform u, winter w) on an index of potential opportunities for *Callitris glaucophylla* regeneration in summer from 1950 to 2008 (*R*^2^ = 0.93).

**Table:**

Source	SS	df	MS	*F*	*P*
Corrected model	79.60	5	15.92	138.21	<0.001
Intercept	0.11	1	0.11	0.98	0.327
Annual rainfall (mm yr^−1^)	45.32	1	45.88	393.44	<0.001
Seasonal rainfall (s, u, w)	0.79	2	0.39	3.44	<0.001
Annual × seasonal rainfall	0.27	2	0.14	1.18	0.315
Error	5.76	50	0.12		
Total	978.00	56			
Corrected total	85.37	55			

The index was square root transformed.

## Appendix 4

Results of proportion tests used to examine the occurrence of disturbances with mean annual rainfall (<405 mm, >405 mm) and seasonal rainfall (summer s, winter w, uniform u).

**Table:**

Disturbance	Mean annual rainfall (mm)				Seasonal rainfall zone			
	
	*X*^2^	df	*P*	Directionality	*X*^2^	df	*P*	Directionality
Farms	13.80	1	0.002	Below > above	3.37	2	0.190	
Rabbits	10.20	1	0.001	Below > above	6.86	2	0.030	w > s
Sheep	3.91	1	0.048	Below > above	0.41	2	0.810	
Cattle	1.31	1	0.250		1.49	2	0.480	
Goats	1.31	1	0.290		9.61	2	0.008	u > w
Kangaroos	0.04	1	0.840		0.11	2	0.950	

Fire was not statistically analyzed.
